# Mapping environmental suitability of *Haemagogus* and *Sabethes spp*. mosquitoes to understand sylvatic transmission risk of yellow fever virus in Brazil

**DOI:** 10.1371/journal.pntd.0010019

**Published:** 2022-01-07

**Authors:** Sabrina L. Li, André L. Acosta, Sarah C. Hill, Oliver J. Brady, Marco A. B. de Almeida, Jader da C. Cardoso, Arran Hamlet, Luis F. Mucci, Juliana Telles de Deus, Felipe C. M. Iani, Neil S. Alexander, G. R. William Wint, Oliver G. Pybus, Moritz U. G. Kraemer, Nuno R. Faria, Jane P. Messina

**Affiliations:** 1 School of Geography and the Environment, University of Oxford, Oxford, United Kingdom; 2 Departamento de Ecologia, Instituto de Biociências, Laboratório de Ecologia de Paisagens e Conservação—LEPAC, Universidade de São Paulo, São Paulo, Brazil; 3 Department of Pathobiology and Population Sciences, Royal Veterinary College London, London, United Kingdom; 4 Centre for the Mathematical Modelling of Infectious Diseases, London School of Hygiene & Tropical Medicine, London, United Kingdom; 5 Department of Infectious Disease Epidemiology, Faculty of Epidemiology and Population Health, London School of Hygiene & Tropical Medicine, London, United Kingdom; 6 State Centre of Health Surveillance, Rio Grande do Sul State Health Secretariat, Rio Grande do Sul, Brazil; 7 MRC Centre for Global Infectious Disease Analysis, Department of Infectious Disease Epidemiology, Imperial College London, London, United Kingdom; 8 Superintendence for Endemic Diseases Control, São Paulo State Health Secretariat, São Paulo, Brazil; 9 Fundação Ezequiel Dias—Lacen/MG, Minas Gerais, Brazil; 10 Environmental Research Group Oxford, c/o Department of Zoology, University of Oxford, Oxford, United Kingdom; 11 Department of Zoology, University of Oxford, Oxford, United Kingdom; 12 Departamento de Molestias Infecciosas e Parasitarias & Instituto de Medicina Tropical da Faculdade de Medicina da Universidade de São Paulo, São Paulo, Brazil; 13 Oxford School of Global and Area Studies, University of Oxford, Oxford, United Kingdom; University of Hong Kong, HONG KONG

## Abstract

**Background:**

Yellow fever (YF) is an arboviral disease which is endemic to Brazil due to a sylvatic transmission cycle maintained by infected mosquito vectors, non-human primate (NHP) hosts, and humans. Despite the existence of an effective vaccine, recent sporadic YF epidemics have underscored concerns about sylvatic vector surveillance, as very little is known about their spatial distribution. Here, we model and map the environmental suitability of YF’s main vectors in Brazil, *Haemagogus spp*. and *Sabethes spp*., and use human population and NHP data to identify locations prone to transmission and spillover risk.

**Methodology/Principal findings:**

We compiled a comprehensive set of occurrence records on *Hg*. *janthinomys*, *Hg*. *leucocelaenus*, and *Sabethes spp*. from 1991–2019 using primary and secondary data sources. Linking these data with selected environmental and land-cover variables, we adopted a stacked regression ensemble modelling approach (elastic-net regularized GLM, extreme gradient boosted regression trees, and random forest) to predict the environmental suitability of these species across Brazil at a 1 km x 1 km resolution. We show that while suitability for each species varies spatially, high suitability for all species was predicted in the Southeastern region where recent outbreaks have occurred. By integrating data on NHP host reservoirs and human populations, our risk maps further highlight municipalities within the region that are prone to transmission and spillover.

**Conclusions/Significance:**

Our maps of sylvatic vector suitability can help elucidate potential locations of sylvatic reservoirs and be used as a tool to help mitigate risk of future YF outbreaks and assist in vector surveillance. Furthermore, at-risk regions identified from our work could help disease control and elucidate gaps in vaccination coverage and NHP host surveillance.

## Author summary

Yellow fever virus (YFV) is an arbovirus transmitted to humans from mosquitoes and can lead to severe disease and death. Recent sporadic outbreaks coupled with low vaccination coverage have highlighted the importance of mosquito surveillance for preventing future outbreaks and potential virus spillover into dense urban areas. Yet, very little is known about the spatial distribution of mosquitoes known to transmit YFV and the factors that contribute to their environmental suitability in Brazil. We compiled an occurrence database of primary and secondary mosquito vectors belonging to *Haemagogus* and *Sabethes* species’ collected between 1991–2019 and integrated this data with environmental and land-use data to predict their spatial suitability at 1 km x 1 km resolution. Using this information, we identified suitable regions for their co-existence. We overlaid this information with human population density and locations of non-human primate host reservoirs to identify areas at risk of transmission and spillover. Our study provides high-resolution mapping tools to assist with mosquito and arbovirus surveillance which is especially useful in low-resource settings.

## Introduction

In Brazil, several sylvatic mosquito species’ belonging to the genera *Haemagogus* and *Sabethes* are the primary and secondary vectors of yellow fever virus (YFV) [1,2]. YFV is an arbovirus belonging to the family *Flaviviridae* that causes acute infectious disease in humans. Literature states that among non-vaccinated populations from Africa and South America, about 55% (95% credible interval, CI: 37%-74%) of individuals infected with YFV are asymptomatic, while about 33% (95% CI: 13%-52%) develop mild disease. Among the approximate 12% (95% CI: 5%-26%) that develop severe disease, case fatality can reach 47% or greater (95% CI: 31%-62%) [3]. In peri-urban and urban areas, transmission of YFV can also be maintained between *Aedes aegypti* (Linnaeus, 1972) and humans; however, this transmission cycle has not been documented in Brazil since 1942 [4]. YFV is endemic in the Brazilian Amazon Basin and mainly remained there until the 1940s, when sporadic outbreaks were observed in the Northeast, Southeast, and Southern states [4–6]. Recent re-emergence of YFV in Southeastern Brazil between 2016 and 2018 caused the worst YF outbreaks in the last 70 years [7].

Despite the existence of an effective YF vaccine since 1937, eradication efforts in Brazil have been undermined by an enzootic sylvatic transmission cycle maintained in rural areas by reservoirs of *Haemagogus spp*. and *Sabethes spp*. belonging to the Culicidae family. These species feed on non-human primates (NHP) (e.g. *Alouatta sp*.) that dwell in high canopy forests, and occasionally on humans that live in or travel to these areas. During the 2016–2018 outbreaks, *Hg*. *janthinomys*, which can be naturally infected with YFV [8], was found to be the primary vector, while infected *Hg*. *leucocelaenus* were also abundant in outbreak areas [1]. *Sa*. *chloropterus* was found to play a local or secondary role in sylvatic transmission due to limited abundance and distribution [1]. These mosquitoes are vertically dispersed in forest areas [9] and naturally prefer to bite and breed at high canopy levels [10,11]. Due to environmental disturbances, contact between sylvatic mosquitoes and human populations have increased [12]. While the occurrence of YF cases follows a seasonal pattern, during which most cases are reported between December to May, outbreaks are still highly dependent on environmental conditions that facilitate transmission [7]. Given the frequency and severity of recent epidemics and the ubiquity of human travel to sylvatic transmission zones, there is increasing concern for a sylvatic-to-urban cycle spillover into *Ae*. *aegypti* infested areas with low vaccination coverage, especially those in near proximity to sylvatic environments. Thus, a greater understanding of the distribution of sylvatic vectors of YFV and its implications for a spillover is needed to help prevent future epidemics.

Species distribution modelling (SDM) techniques are useful for mapping the distribution of arbovirus vectors by focusing on their ecology as predicted from a combination of data on climate, environmental, and population factors [13,14]. To date, only two studies have attempted to map the distribution of sylvatic vectors of YFV. Childs et al. [15] applied SDM using a MaxEnt model to determine the combined distribution of *Hg*. *janthinomys*, *Hg*. *leucocelaenus*, and *Sa*. *chloropterus* in Brazil, by integrating data on climatic and environmental factors and occurrence data retrieved from the Global Biodiversity Information Facility (GBIF) and published literature. Similarly, Almeida et al. [16] predicted the distribution of *Hg*. *leucocelaenus* in the state of Rio Grande do Sul using occurrence data from the 2008–2009 epizootics.

However, these results alone may not accurately determine the ecological niche of each vector and their locations given use of confined occurrence data and limited presumptions about the environmental conditions that influence their distribution in Brazil. Random sampling assumptions adopted for identifying their potential absence via the MaxEnt model may reflect unbiased surveillance efforts, which is rarely the case in the field. While Childs et al. [15] adopted a pseudo-absence selection method consisting of subsampling from occurrence data on mosquito species other than *Hg*. *janthinomys*, *Hg*. *leucocelaenus*, and *Sa*. *chloropterus*, this method may incorrectly identify potential pseudo-absence locations as it classifies excluded species of *Haemagogus* and *Sabethes* sharing similar ecology with the aforementioned primary vectors as pseudo-absences. Given that little is known about these vectors and their niches, these limitations should be carefully considered. Moreover, neither study assessed the environmental suitability of *Hg*. *janthinomys*, *Hg*. *leucocelaenus*, and *Sabethes spp*. individually across Brazil, which is important given differences in their ecological niche and distributions across space.

Building on these previous efforts, we modelled the distribution of *Hg*. *janthinomys*, *Hg*. *leucocelaenus*, and *Sabethes spp*. in Brazil by compiling the most comprehensive dataset of *Haemagogus* and *Sabethes spp*. to date, which contains not only occurrence data but also records from GBIF database, published literature, and primary entomological surveys collected between 1991 and 2019. Using covariate data representative of environmental conditions (daytime land surface temperature, precipitation, relative humidity, and land-cover classes) and human activity (population density and deforestation) conducive to sylvatic mosquito activity (as confirmed by lab and field studies), we modelled the probability of environmental suitability for each species using a stacked regression ensemble approach consisting of three different models to increase predictive performance. By combining the resulting predicted species suitability from this study with modelled presence probability of non-human primate host reservoirs, within and surrounding the Southeastern Atlantic forest biome [17], we further mapped the risk of YFV transmission and spillover in highly populated areas of the region. These maps could inform policy makers the spatial distribution of current and emerging YFV risk zones, which can be targeted for transmission prevention and vaccination programmes.

## Materials and methods

We adopted a stacked binomial regression ensemble modelling approach to quantify the probability of environmental suitability of *Hg*. *janthinomys*, *Hg*. *leucocelaenus*, and *Sabethes spp*., respectively. A stacked regression ensemble is a diverse combination of base models (level-0), with potentially different assumptions about the predictions, into a meta-model (level-1), which has equal or better predictive performance than each individual model [18]. The meta-model is trained on predictions made by the base models and is adjusted based on an optimal weighted average of these models. Previously, this ensemble approach was adopted to map *Plasmodium falciparum* malaria parasite prevalence in sub-Saharan Africa, and was found to outperform any individual method [19]. Here we used three models in an ensemble: a lasso or elastic-net regularized generalized linear model (GLM), an extreme gradient boosted regression tree model, and a random forest model. We chose these models because they were suitable for binary prediction and have relatively good generalizability for disease mapping [20,21].

**Fig 1:**
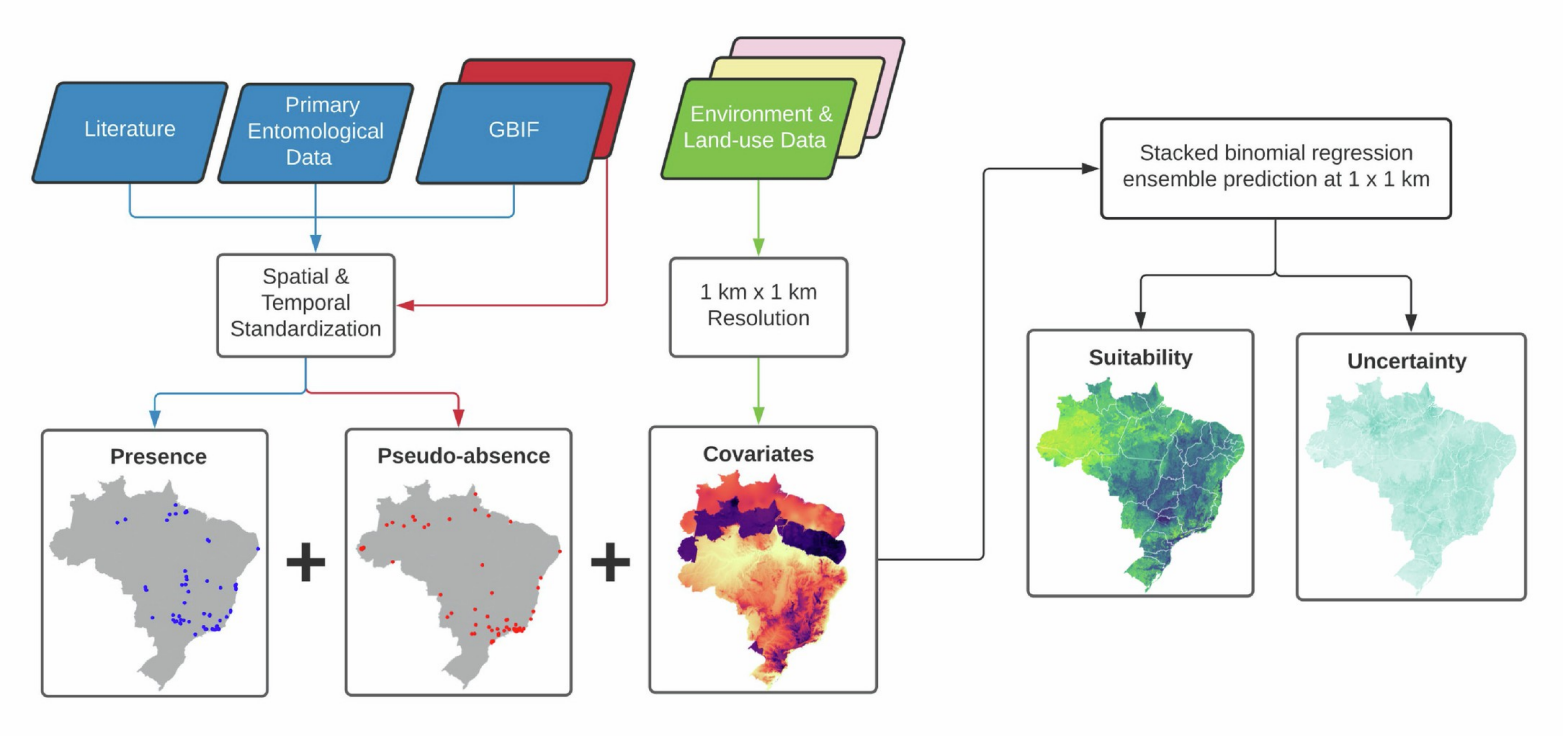
Schematic diagram of the modelling approach adopted to produce maps of environmental suitability for *Haemagogus spp*. and *Sabethes spp*. at 1 km x 1 km resolution. The base layer of the map was retrieved from https://www.ibge.gov.br/geociencias/downloads-geociencias.html.

We used the following set of input variables to run the ensemble: (i) a comprehensive dataset of geocoded occurrence locations for *Hg*. *janthinomys*, *Hg*. *leucocelaenus*, and *Sabethes spp*. recorded from 1991–2019; (ii) selected environmental and land-cover covariate datasets that help to explain the ecological niche of each species; and (iii) a dataset outlining the geocoded locations of pseudo-absence records which refines the geographical range of species occurrence and reduces sampling bias [21]. This is described in more detail in the following sections and in S1 and S2 Figs and S1 Table, alongside full details of the data and each analysis stage. An outline of the methodology from data assembly, modelling approach, to outputs is described in Fig 1.

### Presence records

To assess contemporary distributions, we extracted all records of *Hg*. *janthinomys*, *Hg*. *leucocelaenus*, and *Sabethes spp*. between the years 1991–2019 from our compiled dataset, which contains the geographical coordinates of species occurrence. Due to sparse records, we extracted all species of *Sabethes spp*. that have been confirmed of transmitting, capable of being naturally infected, or are found before and during previous and recent YFV outbreaks in Brazil. This includes *Sa*. *chloropterus*, *Sa*. *albiprivus*, *Sa*. *soperi*, *Sa*. *glaucodaemon*, *Sa*. *cyaneus*, *Sa*. *purpureus*, *Sa*. *melanonymphe*, *Sa*. *undosus/fabrici/ignotus*, *Sa*. *intermedius*, *Sa*. *belisarioi*, *Sa*. *aurescens*, *Sa*. *identicus*, *Sa*. *xyphydes*, and *Sabethes sp*. [1,2,8,11]. To our knowledge, our occurrence dataset (S1 Appendix) is the most comprehensive for these species in Brazil to date, which contains the geocoded information on the occurrence of pupae, larvae or eggs, and adults of each species recorded between 1922–2019 in Brazil.

**Fig 2:**
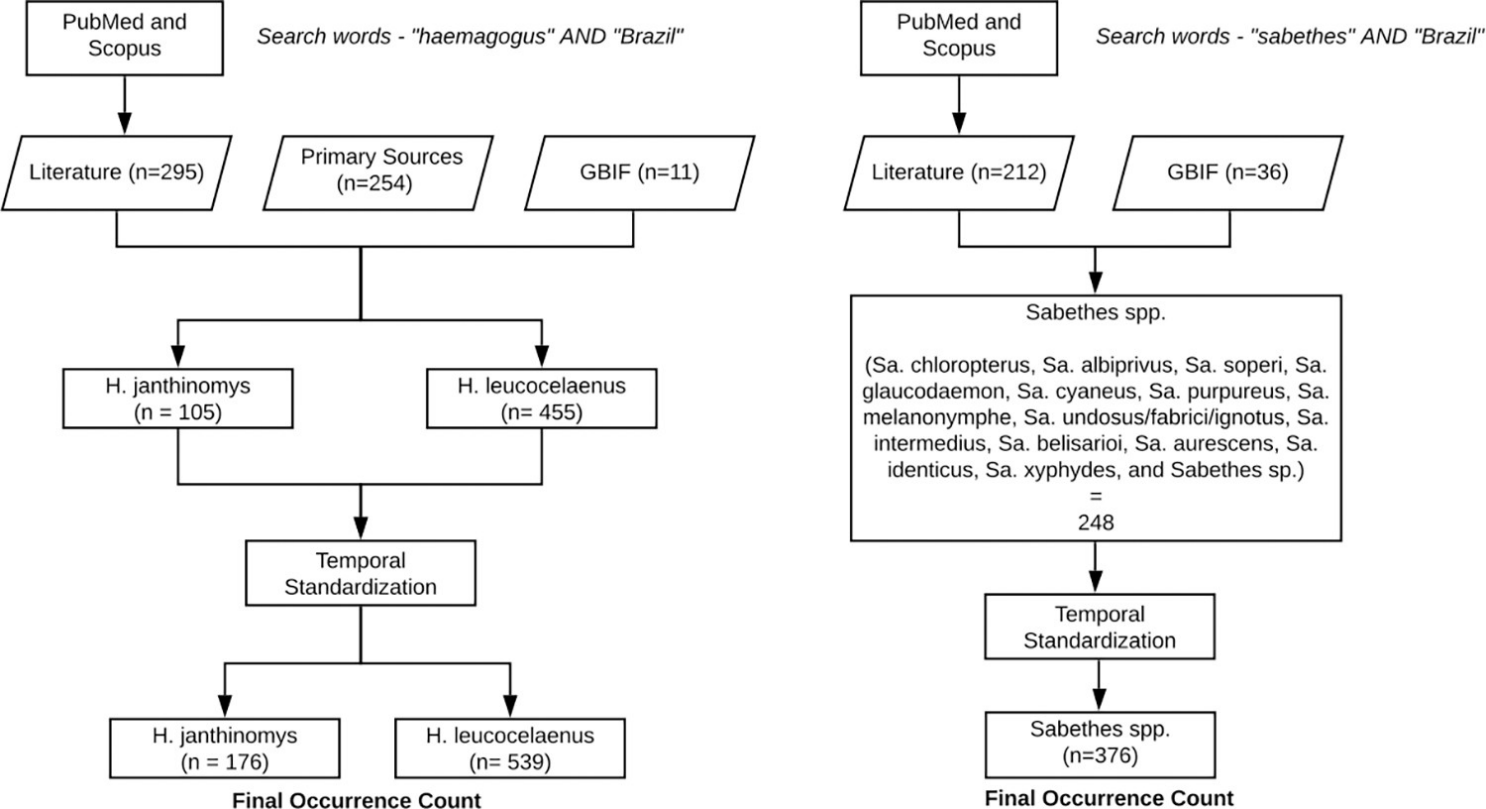
Number of occurrence records for *Hg*. *janthinomys*, *Hg*. *leucocelaenus*, *and Sabethes spp*. (1991–2019) prior and post standardization.

The sources of these data were from (i) The Global Biodiversity Information Facility (GBIF; https://www.gbif.org/) database, (ii) published literature (see list of papers in occurrence data set), and (iii) primary occurrence data from entomological surveys collected by the Health Secretariat of the State of Rio Grande do Sul and Projeto Cantareira as part of São Paulo State Superintendence for Endemic Diseases Control (SUCEN). To retrieve occurrences from the literature, we conducted searches on Scopus and PubMed using the search strings “haemagogus AND Brazil” and “sabethes AND Brazil” (Fig 2). We used the geographical coordinates (latitude and longitude) of each observed occurrence when available. In cases where only an area location was mentioned (e.g. name of town or city), we used Google Maps to extract the coordinates of the centroid of the cited location. The number of records retrieved by year is presented in the S1 Fig. To permit reproducibility, all occurrence data containing the aforementioned information are made freely accessible online on https://github.com/sabrinalyli/YFVMappingBrazil.git.

We performed spatial and temporal standardization using methods described elsewhere [14,22]. This creates a list of occurrences in which each occurrence is individually linked with a unique location and year. This ensures that occurrences from regions where multiple surveys are conducted are not overly-represented compared to occurrences from less well sampled regions. To validate the geo-positioning of occurrence locations, we overlaid the occurrence points with a map of Brazil to ensure that points were geocoded to land only. Point locations that were incorrectly geocoded to a location over water were adjusted using an algorithm described elsewhere [23] or manually checked for validity.

### Environment and land-use variables

Data on the spatial and temporal distribution of environmental conditions are extracted from high-resolution satellite raster imagery. We standardized all covariates to a 1 km x 1 km spatial resolution by resampling each raster using the bilinear method [24] and have outlined the rationale for inclusion in our models below. A summary of metadata for our covariate datasets along with details of covariate sensitivity analysis can be found in S1 Table and S3 Fig. All data processing was done in R (Vers. 4.0.2) using the *rgdal, raster*, and *sf* packages.

### Temperature, precipitation, and relative humidity

Climatic factors such as temperature, relative humidity, and precipitation influence the abundance and activity of *Haemagogus* and *Sabethes spp*. [25–27]. These mosquitoes exhibit daytime biting behaviour [28,29]; in particular, *Hg*. *leucocelaenus* only travel closer to the ground to feed during the day [30]. To account for temperature in our model, we adopted mean annual daytime land surface temperature layers extracted from MODIS v6 time series processed using the Temporal Fourier Analysis (TFA) described elsewhere [31]. For precipitation, we calculated yearly mean total precipitation, yearly minimum precipitation, and yearly maximum precipitation, averaged between years 2001–2019 using data retrieved from ERA5 dataset provided by the European Centre for Medium-Range Weather Forecasts (ECMWF). Minimum annual relative humidity from 2015 was determined as a percentage of water vapour needed to saturate the air given surrounding temperature [32], and was extracted for each 1 km x 1 km pixel.

### Enhanced Vegetation Index (EVI) and vegetation type

*Haemagogous* and *Sabethes* species have an affinity for forest canopies and inhabit tree holes, bamboo nodes, bromelias, and coconut husks in vegetated areas [33,34]. Many complete their larval development in temporarily flooded forest areas [35]. *Haemagogus* and *Sabethes spp*. are primarily found in rain forests and deciduous forests [25,36]. To characterize vegetation cover, we used EVI retrieved from MODIS, a measure of vegetation canopy greenness ranging from 0–1, where values near 1 indicate lush and dense vegetation. We also included consensus land-cover layers processed by Tuanmu et al. [37] detailing the prevalence of evergreen and deciduous trees.

### Forest cover and deforestation

Sylvatic mosquito abundance could be influenced by increasing deforestation activities as a result of regular landscape transformation to accommodate agricultural activities and cattle grazing in recent years [4,38]. To characterize forest cover and deforestation, we adopted gross forest cover loss for the years 2000–2019, using data processed by Hansen et al. [39]. We also included a consensus land cover on cultivated and managed vegetation to account for land that has been anthropogenically modified for agricultural purposes.

### Elevation

While *Haemagogus* and *Sabethes spp*. are known to prefer canopy habitats within the forest strata, they can also be found near the ground depending on the time of the year and climate conditions [11,40,41]. Thus, to account for vertical oviposition, we extracted minimum elevation in meters above sea level from a raster processed by Amatulli et al. [42].

### Population density

Higher population density in cities has the potential to increase physical contact between people and YFV mosquito vectors, which could potentially instigate and maintain another urban YFV transmission pathway in Brazil [43]. Furthermore, including population density in our model allows us to account for the possible occurrence of *Hg*. *janthinomys* and *Hg*. *leucocelaenus* in forest-city ecotones. To account for population density, we included population density estimates adjusted to match country estimates reported by the United Nations from WorldPop (https://www.worldpop.org/) calculated using methods described elsewhere [44].

### Non-human primate reservoirs

We retrieved the presence probability of NHP reservoirs predicted at 1 km x 1 km resolution for six species within the Southeastern Atlantic forest in Brazil, which was determined using occurrence records from 1992–2016 retrieved from Culot et al. [17]. Methods for producing these rasters are described elsewhere [45]. The six species of monkeys were of the following genera: *Alouatta sp*., *Brachyteles sp*., *Callicebu*s sp., *Callithrix sp*., *Leontopithecus sp*., and *Sapajus sp*. These species have been found to host and transmit YFV in Brazil [46,47]. In particular, Howler monkeys (*Alouatta sp*.*)* are extremely susceptible to YFV and can develop fatal disease [48].

### Modelling approach

Our stacked binomial regression ensemble consists of an elastic-net regularized GLM, extreme gradient boosted regression trees, and random forest. An elastic-net regularized GLM can be used as a binary classifier that adopts regularization to shrink the coefficients of the linear model to reduce overlearning and to allow for better generalization [49]. Both extreme gradient boosted regression trees and random forest use an ensemble of regression trees to increase prediction accuracy [50]. The method of gradient boosting builds trees in a stagewise process, where new trees help to correct errors created by the previous tree, while random forest trains each tree independently using a randomly sampled training dataset that makes the model more robust and less prone to overfitting [50]. Boosted regression trees, first described for disease mapping in Elith et al. [51], have been adopted to map several arboviral diseases and vectors [52–54]. The method has also been previously adopted to estimate environmental suitability of *Aedes* vectors known to facilitate arbovirus transmission [14,55]. Within the ensemble, we adopted extreme gradient boosting, which is designed to be computationally efficient with better model performance [56].

From the ensemble, we retrieved a predicted probability of suitability at 1 km x 1 km spatial resolution. To obtain uncertainty estimates around the prediction, we fitted an ensemble of 100 sub-models by applying a separate bootstrap sampling of the dataset for each model. We then determined the distribution of pixel-based uncertainty by calculating the 95% confidence interval derived from the 100 sub-models. All data analysis were conducted using raster, gbm, dismo, and seegSDM packages in R (Vers. 4.0.2). All models were fitted using the *Superlearner*, *glmnet*, *xgboost*, and *ranger* packages in R.

### Pseudo-absence records for removing sample selection bias

A sample of background data defined by absence records is required by BRT models to understand conditions available to the modelled species in an area. Phillips et al. [21] proposed a pseudo-absence selection approach using background records that share the same environmental characteristics as the presence records. This minimizes sample selection bias while highlighting the differentiation between the distribution of presence records and that of the background. We adopted this method by compiling a pseudo-absence dataset containing records of similar acrodendrophilic mosquitoes that belong to the same Culicidae family and are captured using the same methods and equipment in the field. A total number of 1,056 records including *Culex spp*., *Limatus spp*., *Wyeomyia spp*., *Psorophora spp*., *Orthopodomyia spp*., *Mansonia spp*., and *Coquillettidia spp*. observed during 1992–2020 were extracted from the GBIF database (S1 Appendix). The locations of these records are shown in S2 Fig.

### Mapping suitable areas, risk of transmission, and risk of spillover

To better visualize areas that are suitable for vector species, we mapped predicted suitability above a certain threshold. This was done by highlighting pixels where predicted suitability was greater than 0.5 for each species and for all species. We also conducted a sensitivity analysis by changing the threshold to 0.25 and 0.75. Using these data, we mapped the risk of transmission and risk of spillover from sylvatic environments by determining areas of overlap between two to three variables: high environmental suitability of vector species, high predicted presence probability of NHPs, and presence of human population (yes/no) based on population density. High environmental suitability was defined by suitable areas filtered by the threshold. Likewise, high predicted presence probability of NHPs was defined by a probability greater than 0.5.

We defined the risk of transmission by the presence of two variables, which interact to maintain transmission. Firstly, a transmission cycle can be maintained by the presence of sylvatic mosquito vectors and NHP hosts. Secondly, although YF transmission can be maintained by *Aedes spp*., it was not observed during the 2016–2018 outbreaks. Nonetheless, increasing overlap between human populations and NHP host reservoirs could instigate an urban cycle. Lastly, the presence of human populations in areas where sylvatic vectors are present can also foster transmission. Similarly, we defined the risk of spillover by the presence of all three variables in an area, where local transmission is maintained by the presence of mosquito vectors and NHP hosts that feed on nearby human populations. Finally, areas where only one variable is present are defined as low risk. Despite the lack of overlap, transmission could still occur if humans, NHP hosts, and mosquito vectors are assumed to be mobile within given suitable environmental conditions and reasonable travel behaviour.

### Model validation

We calculated the overall predictive accuracy of the model ensemble by quantifying the area under the curve (AUC) statistic and its 95% confidence interval. This was done by running our model ensemble on a randomly sampled subsection of the data referred to as the training set of the data, which contains 80% of the original data. Cross-validation on each model was performed by evaluating the mean area under the curve (AUC) statistic from 10 separate cross-validation folds. This model was then applied for prediction on the holdout set (20% of the original data). Using 2000 bootstraps, we computed the 95% confidence interval. All analyses were performed using the *ROCR* [57] and *pROC* [58] packages in R.

All covariates used in the model were assessed for their variable importance using the permutation method [59,60], which is measured as a change in the model error. Values of each covariate in the data were randomly permuted and assessed for its effect on the model performance using the difference between a baseline performance measure (e.g. RMSE) before and after permutation. This was performed on the stacked binomial regression ensemble using the *vip* package in R [61].

## Results

**Fig 3:**
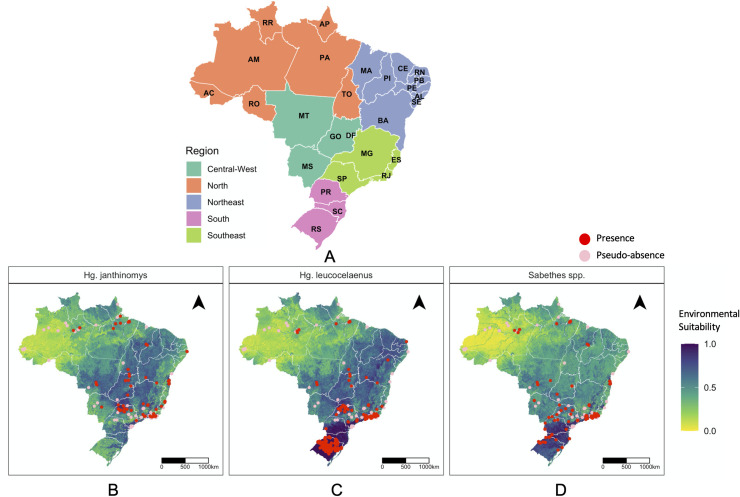
Environmental suitability and occurrence records of *Haemagogus* and *Sabethes spp*. **A,** Map of Brazil by states (AC–Acre, AL–Alagoas, AP–Amapá, AM–Amazonas, BA–Bahia, CE–Ceará, DF–Distrito Federal, ES–Espírito Santo, GO–Goiás, MA–Maranhāo, MT–Mato Grosso, MS–Mato Grosso do Sul, MG–Minas Gerais, PA–Pará, PB–Paraíba, PR–Paraná, PE–Pernambuco, PI–Piauí, RJ–Rio de Janeiro, RN–Rio Grande do Norte, RS–Rio Grande do Sul, RO–Rondônia, RR–Roraima, SC–Santa Catarina, SP–São Paulo, SE–Sergipe, TO–Tocantins) and regions (colour-coded). **B-D,** Maps indicating the point locations (red) of standardized occurrence records of each species collected between 1991–2019 and predicted environmental suitability for *Hg*. *janthinomys*, *Hg*. *leucocelaenus*, and *Sabethes spp*. The base layer of the map was retrieved from https://www.ibge.gov.br/geociencias/downloads-geociencias.html.

To characterize the presence of *vectors* we retrieved a total of 1,091 occurrences (*Hg*. *janthinomys* = 176; *Hg*. *leucocelaenus* = 539; *Sabethes spp*. = 376) over the time period 1991–2019 after standardization. Out of all the occurrences recorded for *Hg*. *janthinomys*, most were recorded in the states of Rio de Janeiro (RJ: 27.3%), São Paulo (SP: 17.6%), and Bahia (BA:15.9%) (Fig 3A and 3B) likely due to higher surveillance in recent years as a result of recent outbreaks. For *Hg*. *leucocelaenus*, 55% of all occurrences were recorded in Rio Grande do Sul (RS), while 20.5% and 17.6% were recorded in São Paulo and Rio de Janeiro, respectively (Fig 3C). The distribution of *Sabethes spp*. occurrences adopted in our study were found in São Paulo (27.8%), Rio de Janeiro (19.4%), Rio Grande do Sul (14.4%) and Bahia (8.2%), (Fig 3D).

Fig 3B and 3D also shows the predicted suitability of *Hg*. *janthinomys*, *Hg*. *leucocelaeunus*, and *Sabethes spp*. To evaluate prediction accuracy of the outputs, we determined model performance using the AUC (area under the curve) on our holdout set and found high accuracy (>0.97). While these species are distributed similarly, there are some differences. The Southeastern region part of the Atlantic forest biome encompassing the states of Minas Gerais (MG), Rio de Janeiro (RJ), Paraná (PA), and parts of São Paulo (SP) were predicted to be the most suitable locations for *Hg*. *janthinomys* and *Hg*. *leucocelaenus*, where the species have been recorded in the last three decades. While few species were recorded in these regions, the Central-West Brazilian states incorporating the *Cerrado* biome of Goiás (GO) and Mato Grosso do Sul (MS), along with some areas within Tocantins (TO), Federal District (DF), and Mato Grosso (MT) were also found to be suitable. Highly suitable areas were also predicted in the Northeast state of Bahia and Piauí (PI), and nearby adjacent areas in the states of Ceará (CE), Paraíba (PB), and Pernambuco (PE), where the species have yet to be reported. The Southern states of Rio Grande do Sul (RS), Paraná (PR), and Santa Catarina (SC) were predicted to be highly suitable for both *Hg*. *leucocelaenus* and *Sabethes spp*. For all species, suitability was predicted to be low across the Northern region along the Amazon river basin, where contemporary records of species are sparse. Maps summarizing uncertainty associated with predicted estimates are shown in S4 Fig.

**Fig 4:**
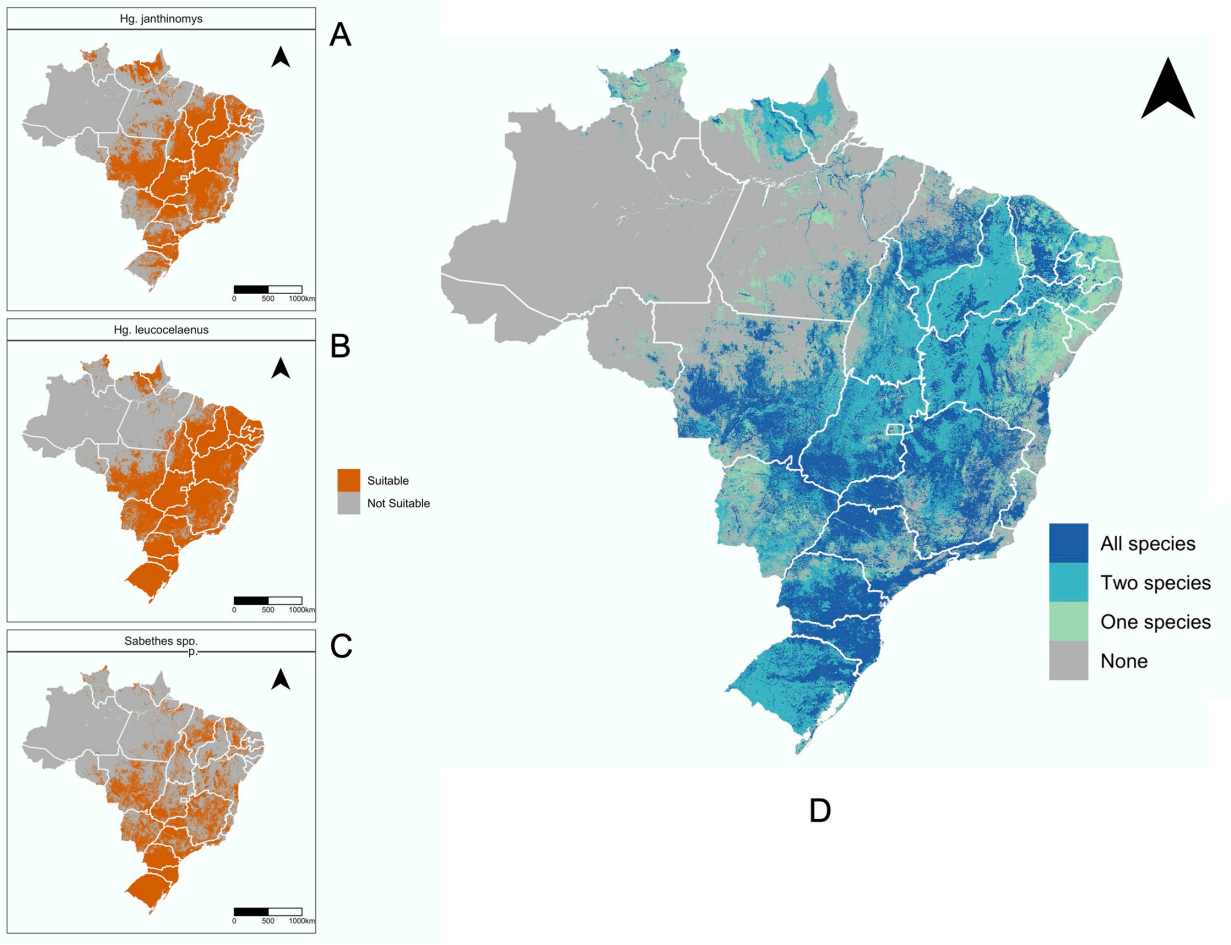
Suitable regions for *Haemagogus* and *Sabethes spp*. **A-C,** Maps of areas identified as suitable with environmental suitability ≥ 0.5. **D,** Map showing areas suitable for only one species, two species, or all species analyzed in this study. The base layer of the map was retrieved from https://www.ibge.gov.br/geociencias/downloads-geociencias.html.

These patterns are further reflected in the maps showing suitable areas (suitability probability ≥ 0.5) for each species (Fig 4A and 4C) and for all species combined (Fig 4D). Our sensitivity analysis shows that 0.5 is an appropriate- threshold to avoid underestimation and overestimation of suitable zones S5 Fig. States that are suitable for all species are mainly concentrated in highly populated areas of the Southeastern region and along part of Northeast and Central-West.

**Fig 5:**
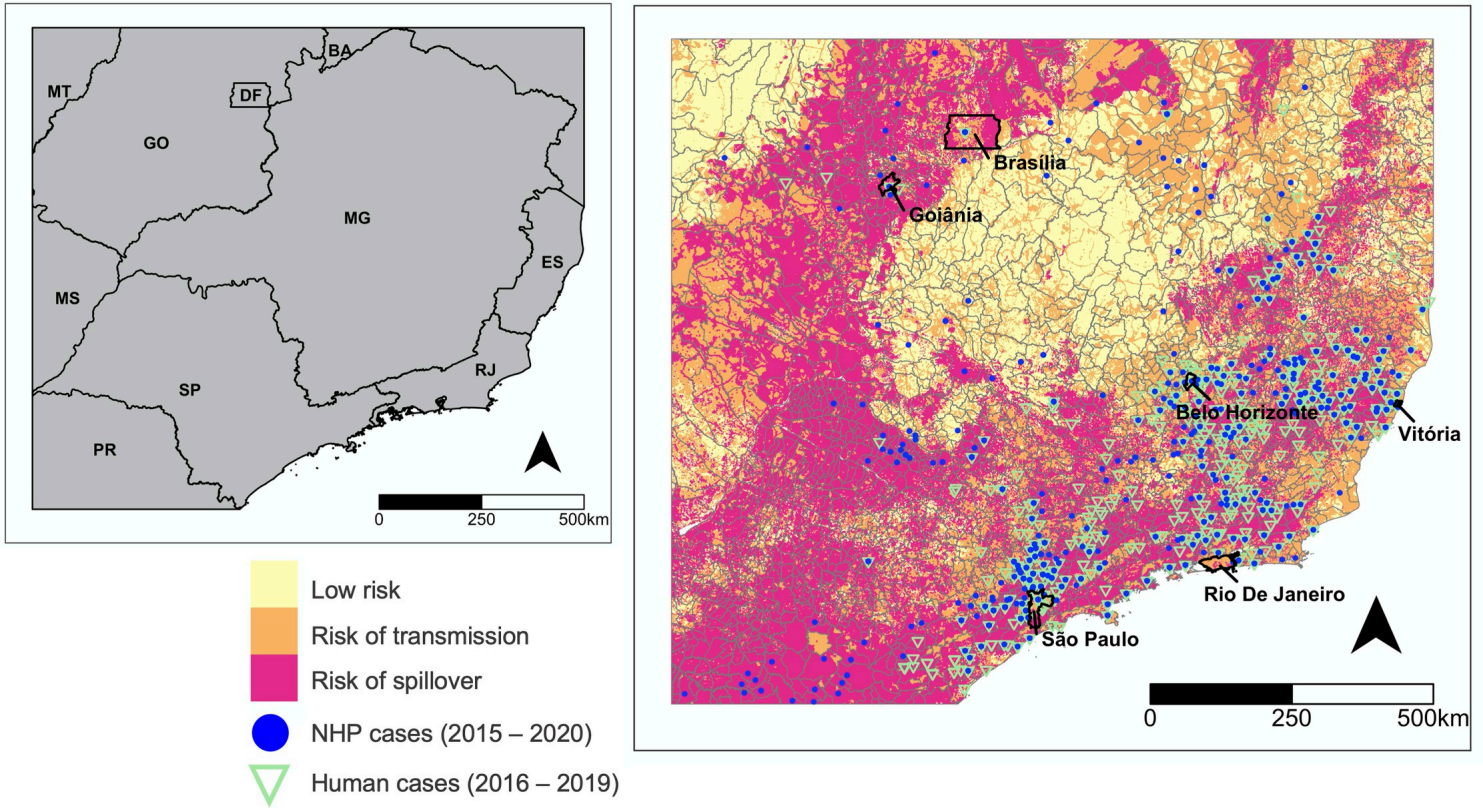
Risk of transmission and spillover along the Southeastern Atlantic Forest Biome. Map showing the distribution in risk status in the Southeastern municipalities of São Paulo, Minas Gerais, Rio de Janeiro, Espírito Santo, and Southern state of Paraná and adjacent municipalities in the Central-West, including the Federal District, Goiás, and Mato Grosso. Risk is defined by the presence of sylvatic mosquito vectors, non-human primate reservoirs, and human population density. Low risk refers to the presence of only one of these factors. Risk of transmission is defined by the overlapping presence of two of these factors, while risk of spillover is defined by the overlapping presence of all three factors. Blue points show the locations of confirmed cases among non-human primates (NHP) recorded between years 2015–2020 while green triangles indicate the locations of confirmed human cases recorded between 2016–2019. The base layer of the map was retrieved from https://www.ibge.gov.br/geociencias/downloads-geociencias.html.

Likewise, our risk map based on the presence of mosquitoes, NHP host reservoirs, and human populations (Fig 5) shows that areas at risk of YFV spillover are concentrated along highly populated municipalities of Paraná, the cities of São José do Rio Preto and São Paulo, as well as municipalities in Rio de Janeiro and Espírito Santo states. Areas at risk of spillover also include the Federal District and Goiás. These areas are surrounded by comparatively less populated municipalities which are at the risk of transmission. The regions between Goiás and Minas Gerais have the lowest risk given the lack of NHP host reservoirs. We superimposed our spillover risk maps with data on confirmed (PCR, immunohistochemistry and/or IgM ELISAs) YFV cases among humans and non-human primates between 2015–2020 [62] retrieved from the Brazilian Ministry of Health National Notifiable Disease System (SINAN; Sistema de Informação de Agravos de Notificação). This shows that previous human and NHP cases clustered mainly in spillover zones. However, the original site of human infection may be different than the site of reporting attributed to passive surveillance coupled with human movement.

Predictors with the strongest variable importance on the model ensemble for *Hg*. *janthinomys* included population density (permuted model error: 0.106±0.001), which shares a negative relationship with species distribution, the prevalence of deciduous tree cover (0.1±0.004), and elevation (0.075±0.001). For modelling *Hg*. *leucocelaenus*, the most influential predictors were elevation (0.097±0.002) and minimum daytime land surface temperature (0.069±0.003), followed by population density (0.067±0.002). Similar results were found for modelling *Sabethes spp*., where the prevalence of deciduous tree cover (0.091±0.002), population density (0.084±0.002), and elevation (0.078±0.002) had the strongest relative influence. Relative to all covariates, mean forest cover loss had consistently one of the least variable importance for all models. Variable importance along with partial dependence plots for *Hg*. *janthinomys*, *Hg*. *leucocelaenus*, and *Sabethes spp* can be viewed interactively from https://github.com/sabrinalyli/YFVMappingBrazil. Overall predictive performance was high for all model ensembles based on computed AUC (S6 Fig).

## Discussion

To understand the spatial distribution and patterns of YFV vectors and their risk to humans, we used a stacked regression ensemble modelling approach to map the environmental suitability of main YFV vectors from the *Haemagogus* and *Sabethes* genera at a high spatial resolution of 1 km x 1 km. This was done using occurrence records reported between 1991–2019 combined with associated environmental and land-cover covariates. Using predicted estimates, we identified suitable regions for each vector and all vectors. We also mapped the distribution of risk of transmission and spillover in regions that overlapped human settlements and the presence probability of NHP host reservoirs.

Previous studies by Childs et al. [15] and Almeida et al. [16] used MaxEnt models with different assumptions about the selection of pseudo-absences. Using a stacked binomial regression model ensemble and pseudo-absence selection based on background records that share the same environmental characteristics as the presence records, we found higher suitability in the Northeast and lower suitability in the North, which is the opposite of the findings presented by Childs et al. [15]. Similar to Almeida et al. [16], our model ensemble also predicted high suitability for *Hg*. *leucocelaenus* in Rio Grande do Sul. Compared to Almeida et al. [16], the integration of additional occurrence points in our model ensemble allowed the prediction of higher suitability for the south of Rio Grande do Sul. Whereas temperature did not play an important role in model prediction in Childs et al. [15], we found it an important covariate in ranking after elevation, population density, and deciduous forest cover.

Despite the existence of effective vaccines, Brazil experienced the largest sporadic YF outbreaks of recent years during 2016–2018, which resulted in more than 2,153 diagnosed human cases and 744 deaths in highly populated urban areas of Southeast Brazil that were previously considered low risk [63]. These outbreaks, which were identified to be of sylvatic origin [1], spilled into the transition zone between the *Cerrado* biome and the Atlantic Forest in Minas Gerais and moved towards the Southeastern states of Minas Gerais, Espírito Santo, Rio de Janeiro, and São Paulo, (61), which corresponds to predicted areas of high environmental suitability for primary and secondary sylvatic vectors in our study. We also identified highly suitable areas in the South and Central-West regions, including Santa Catarina and Rio Grande do Sul, where outbreaks were predicted to occur every six to ten years [4] and have been recently reported for 2019 and 2021 [64,65]. These findings are consistent with recent spatial epidemiological patterns of sylvatic YF outbreaks in the Central-West and Southeastern regions [7,66]. Low predicted suitability in the Amazon river basin is likely due to decreased entomological reporting in recent years, mainly due to lack of surveillance of sylvatic vectors in the Amazon region. These maps can be used as a public health tool and help guide the surveillance of YFV vectors, especially in areas lacking entomological data but were identified as environmentally suitable.

Our understanding of each species’ ecology is confirmed by the relative influence of environmental covariates on the models. The distribution of each species may be dependent on the height of treetop canopies, but further investigation is required. Here we used elevation to characterize vertical oviposition activity. A previous study found that the risk of YFV cases at elevations between 300–800 MASL was six times higher compared to other elevations [67], indicating a potential linkage to sylvatic mosquito activity. Population density strongly influenced species’ distribution via an inverse relationship, corroborating species’ preference for non-urbanized settings. The prevalence of deciduous tree cover had a strong influence on shaping the distribution of both *Hg*. *janthinomys* and *Hg*. *leucocelaenus*. Minimum day-time temperature had a strong influence on both *Hg*. *leucocelaenus* and *Sabethes spp*. species. In particular, higher suitability was detected when temperature was above 27°*C*, which is consistent with recent findings on temperature-population dynamics of *Hg*. *leucocelaenus* [25,68].

Forest loss was found to have an overall weak influence on the models for all three species. Evidence on the relationship between deforestation and mosquito abundance is scarce and remains unclear. In Brazil, *Hg*. *janthinomys* and *Hg*. *leucocelaenus* are highly dispersed between forest areas, and can be found in small forest patches (e.g. 7 hectares) next to urban areas and isolated high-income neighbourhoods located in forest valleys [1], In particular, *Hg*. *leucocelaenus* are often found in modified forest patches near human activities, including isolated forest fragments in cultivated areas and in pasture grasslands generally associated with small rivers and creeks as well as urban green areas [30,69,70]. A systematic review that investigated the effects of deforestation on mosquitoes found that half of mosquito species favoured deforested areas [71]. However the loss of mosquito habitats from the progression of deforestation may also reduce transmission risk [72]. Moreover, some considerations have also been given to the changing agricultural ecosystem in Brazil, which is a main driver of deforestation. Seasonally varying agricultural activities such as planting and harvesting to accommodate the growth of cash crops have been found to capture seasonal YFV reporting in humans and NHPs [38], but little is known about the impacts of regular land conversion through agriculture on mosquito habitats and transmission activity. Nonetheless, more field observations and investigations are needed to understand the effects of agriculture and deforestation on mosquito abundance.

By combining predicted estimates of vector suitability, predicted presence of NHP host reservoirs, and population density, we further characterized and mapped the distribution in risk of YFV transmission and spillover within and surrounding the Southeastern Atlantic Forest biome. Our detection of spillover and transmission risk in and around Minas Gerais, Rio de Janeiro, Espírito Santo, and São Paulo, where previous spillover events have been recorded (62), suggests that disease control and prevention should continue to focus in those areas. Furthermore, our map highlighting spillover risk in Paraná also coincides with the occurrence of epizootics in the state between 2019 and early 2021 [73]. Given that YFV cases among NHPs are recorded through passive surveillance and are thus often based in areas with human settlements, we cannot rule out the fact that varying levels of risk may subsist in non-spillover zones that are not identified on our map. Despite this, these results do not reflect the risk of YFV outbreaks as population level vaccination coverage is not considered. Even if vaccination coverage is considered, the heterogeneity of current vaccination data by municipality may not accurately portray the coverage gradient across space at the local level. Furthermore, we also did not consider the dynamics of human mobility, which also contribute to outbreak risk. Nonetheless, our maps can help guide public health officials in detecting at-risk municipalities, where further investigation can be conducted by comparing vaccination coverage and effectiveness.

In September 2018, only a handful of at-risk municipalities within the states in our study region reached 95% vaccination coverage; this included 13% and 19% of municipalities in Paraná (13%) São Paulo, respectively [74]. However as of May 2020, coverage in the majority of the municipalities in São Paulo, Paraná, Santa Catarina, Espírito Santo, and Minas Gerais was still less than 60% for individuals aged 15 years and older, and less than 80% for individuals aged 14 years and younger [75]. Despite improved coverage, the lack of seroprevalence data for YF presents uncertainty in the disease burden experienced by at-risk populations. A recent seroepidemiological study focusing on the Serro region in Minas Gerais found that about 26% of urban and rural populations did not present neutralizing antibodies against YFV, including some that were vaccinated [76]. In particular, children were less likely to present YFV-neutralizing antibodies and were less likely to have received a booster dose. Furthermore, there has been a reduction in childhood immunization in Brazil from 2017 to 2019 [77]. This highlights potential underestimation of disease burden and the likelihood of new outbreaks originating among vulnerable populations across all ages living in at-risk areas. Moreover, it is uncertain whether current vaccination efforts have been hindered by the co-existence of the COVID-19 pandemic, which may have redirected outbreak surveillance efforts and the allocation of health resources [78].

Our study has a few limitations. It does not account for seasonal patterns of species distribution, which could influence the distribution in environmental suitability of each species throughout the year. Given that the abundance of *Haemagogus* and *Sabethes spp*. is highest between December and May during the rainy season, the risk of transmission and spillover may also vary throughout the year [79]. Despite assembling the most comprehensive database to date, our data comes from a few specific geographic areas and thus may not cover the full range of ecological conditions that influence each species. This could be addressed by limiting predictions to locations outside of model training regions [80] or using a temperature suitability index [81]. Due to limited lab and field evidence on how temperature affects the extrinsic incubation period of these species, their gonotrophic cycle, and oviposition behaviour, future entomological surveys could focus on these observations in various locations to update our knowledge which is mainly based on lab evidence presented from a few decades ago [82–84]. Similarly, while our pseudo-absence records on Culicidae species are assembled from GBIF only, future works could explore the addition of records from literature. While doing so may not overcome the issue of differential surveillance efforts conducted by various research teams, it could potentially minimise selection bias and improve modelled outputs.

Our finding of restricted spatial distribution in environmental suitability for *Sabethes spp*. concur with previous field observations. *Sabethes spp*. was observed in low abundance, distribution, and infection rates and played a local or secondary role during the 2016–2018 outbreaks [1]. Previous studies have noted the competence of *Sa*. *albiprivus*, *Sa*. *petrocchiae*, and *Sa*. *chloropterus* in transmitting YFV [85], however some species are limited to certain ecological conditions. Among the *Sabethes* genera, *Sa*. *chloropterus* was found mainly in forest areas and while considered the primary YFV sylvatic vector [2], was found with low infection rates during previous outbreaks [1,86].

While beyond the scope of this paper, the role of *Aedes spp*. mosquitoes and its interactions with the sylvatic cycle in facilitating YFV transmission should be further investigated given conflicting findings. In Brazil, evidence of YFV transmission from *Ae*. *aegypti* and *Ae*. *albopictus*, which are sensitive to YFV infection, has not been observed since 1942 [1] but other species such as *Ae*. *scapularis* and *Ae*. *taeniorhynchus* were found to be naturally infected during the 2016–2018 outbreaks. While these occasional findings do not define their role as YFV vectors, more investigation is needed on their potential vectorial capacity. In addition, considering human mobility in future studies could also help to identify changes in disease spread facilitated by viremic humans, including those that are asymptomatic, which can be applied to infer the geographical shift in risk over time. Furthermore, current uncertainties related to our knowledge of NHP host species, their viremia, and the spatial distributions of their reservoirs [87] should be further studied. While most NHP exist in sylvatic environments, some are capable of surviving in degraded environments [88]. NHP behaviour of various species across Brazil should be closely monitored to identify reservoir establishment and for epizootics to delimit transmission. In this aspect, the promising application of phylogenetic data and genomic analysis could help to identify the roles of NHP species in facilitating transmission, the variation of YFV infection rates across NHP species, and trends in epizootic cases [89].

In conclusion, our findings expand on our limited understanding on the geographic distribution of potential sylvatic vector reservoirs in Brazil. This work contributes to the growing body of research on vector and vector-borne disease mapping at high spatial resolution in low-and middle-income countries that lack comprehensive surveillance coverage. Our identification of suitable regions for main YFV vectors nationally and areas at risk of YFV spillover in the Southeastern region of Brazil could help guide knowledge users and policy makers working in vector surveillance and vaccine allocation.

## Supporting information

S1 Fig Temporal distribution of occurrence records for *Hg*. *janthinomys*, *Hg*. *leucocelaenus*, and *Sabethes spp*.
(TIFF)

S2 Fig Distribution of pseudo-absence points. The base layer of the map was retrieved from https://www.ibge.gov.br/geociencias/downloads-geociencias.html.
(TIFF)

S3 Fig Maps illustrating the distribution of environmental covariate data. The base layer of the map was retrieved from https://www.ibge.gov.br/geociencias/downloads-geociencias.html.
(TIFF)

S4 Fig (A) Maps of prediction uncertainty calculated based on the 95% confidence interval and (B) frequency of presence occurrence points falling in each predicted range of suitability. The base layer of the map was retrieved from https://www.ibge.gov.br/geociencias/downloads-geociencias.html.
(TIFF)

S5 Fig Maps showing suitable regions for *Haemagogus* and *Sabethes spp*. when environmental suitability threshold is set to (A) ≥ 0.25 and (B) ≥ 0.75. The base layer of the map was retrieved from https://www.ibge.gov.br/geociencias/downloads-geociencias.html.
(TIFF)

S6 Fig AUC and 95% confidence interval generated for the holdout set for *Hg*. *janthinomys*, *Hg*. *leucocelaenus*, and *Sabethes spp*.
(TIFF)

S1 Table Metadata of environmental and land-cover covariates used in stacked binomial regression model.
(DOCX)

S1 Appendix List of occurrence data downloads (with DOI) from Global Diversity Information Facility (GBIF).
(DOCX)
