# Supplementary figures and images for: Mapping environmental suitability of Haemagogus and Sabethes spp. mosquitoes to understand sylvatic transmission risk of yellow fever virus in Brazil
Source: PLoS Negl Trop Dis. 2022 Jan 7;16(1):e0010019. doi: 10.1371/journal.pntd.0010019 (PMC8797211; doi:10.1371/journal.pntd.0010019)

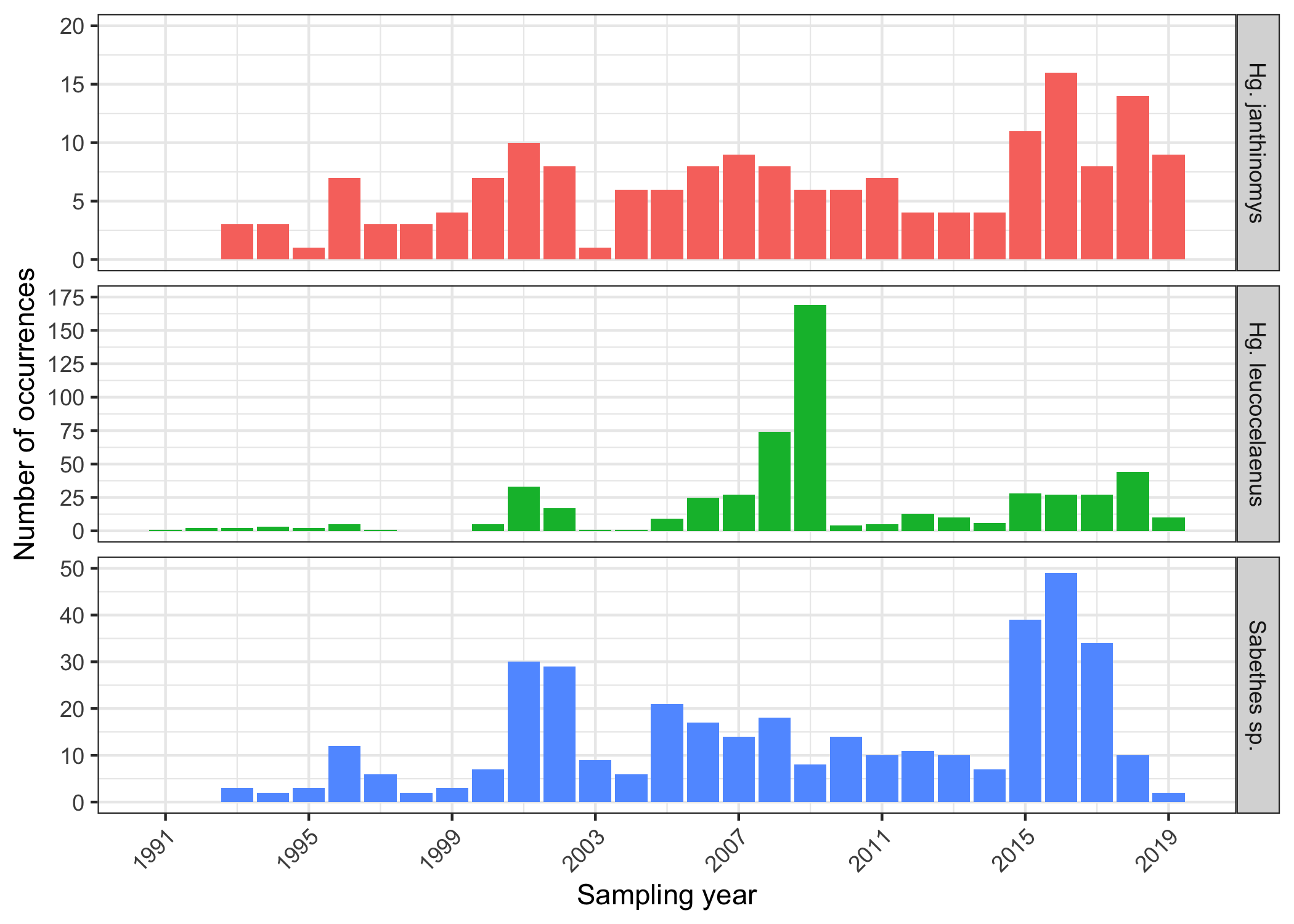

Supplement: S1 Fig — Temporal distribution of occurrence records for Hg. janthinomys, Hg. leucocelaenus, and Sabethes spp. (TIFF) [file pntd.0010019.s001.tiff]

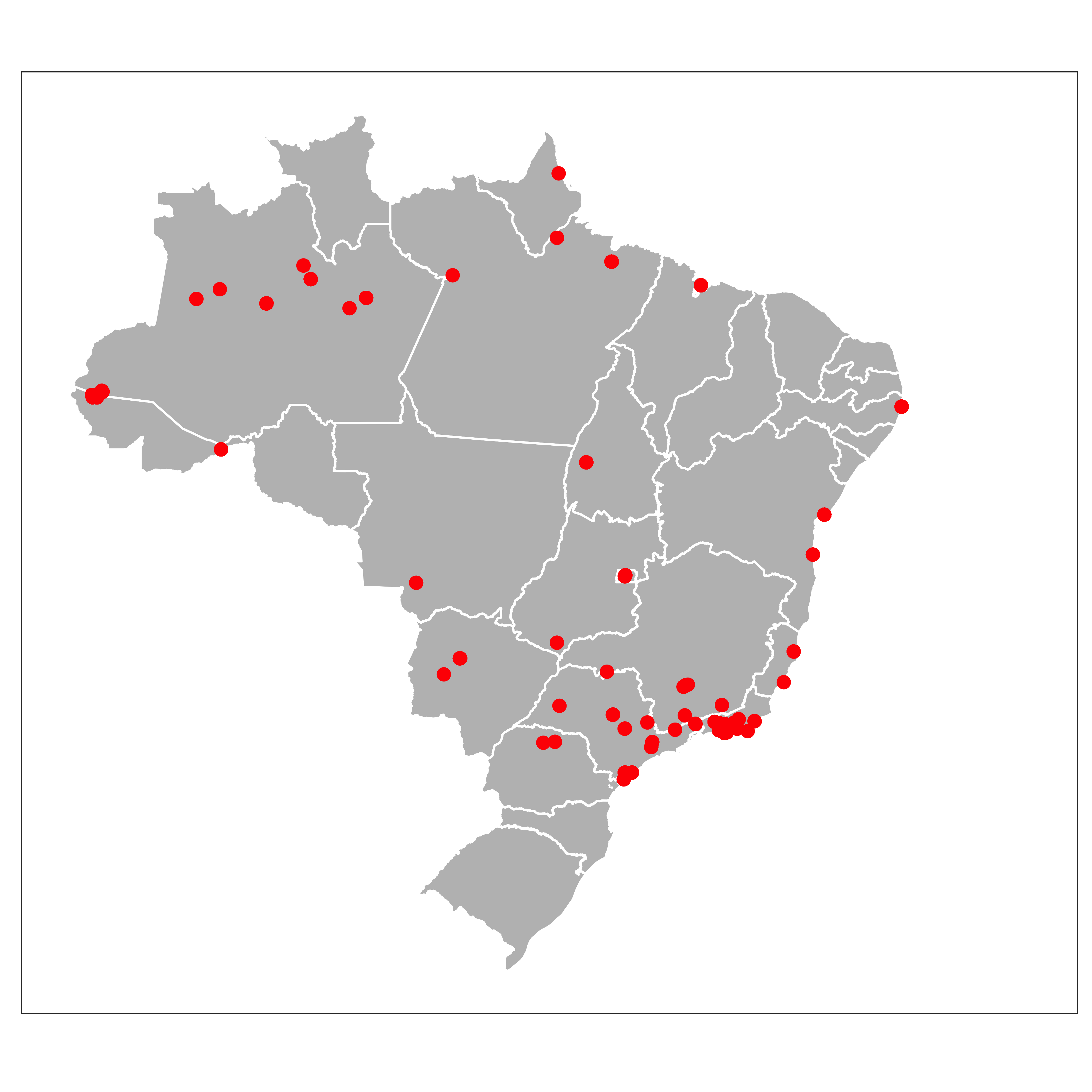

Supplement: S2 Fig — Distribution of pseudo-absence points. The base layer of the map was retrieved from https://www.ibge.gov.br/geociencias/downloads-geociencias.html. (TIFF) [file pntd.0010019.s002.tiff]

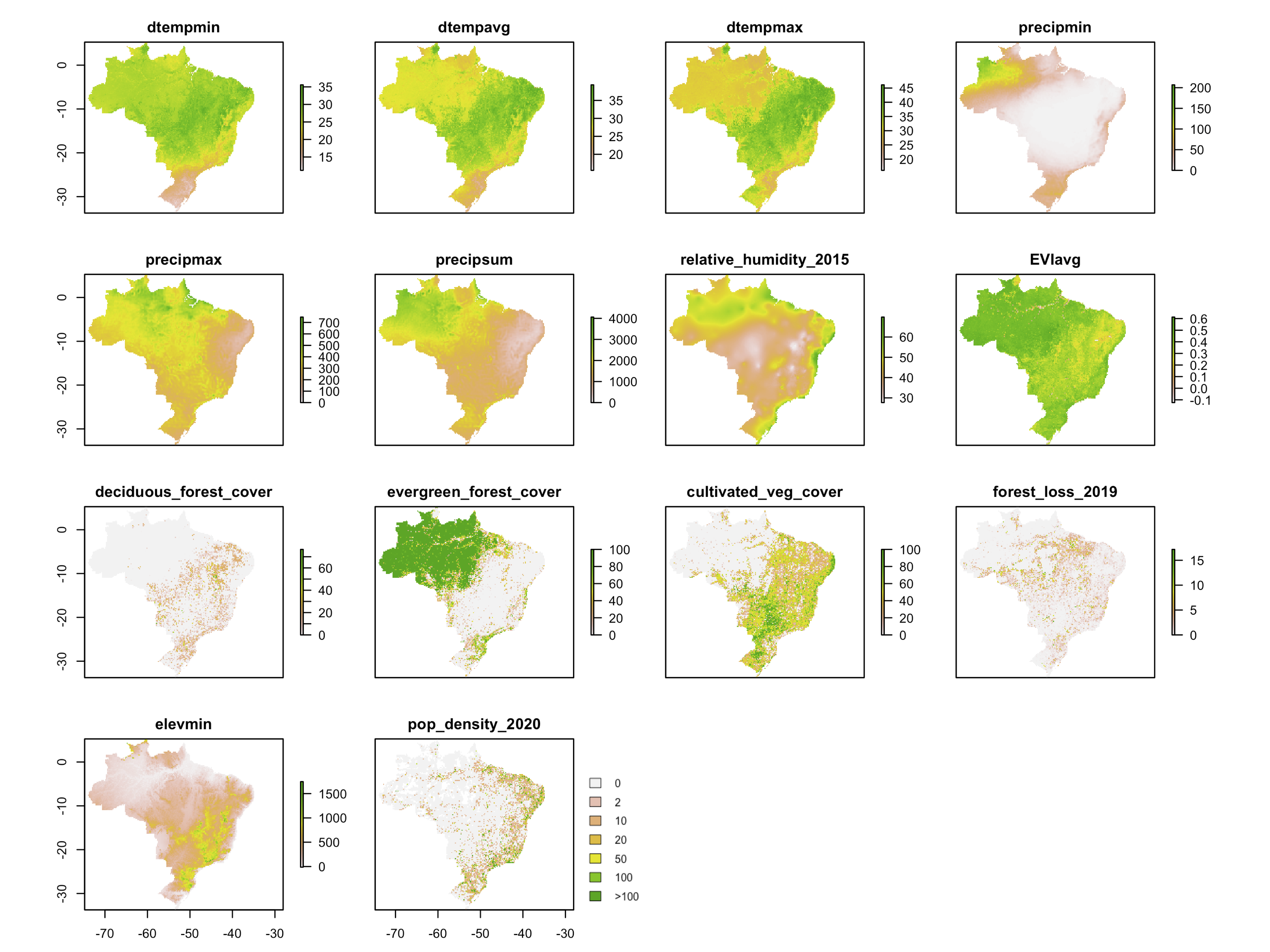

Supplement: S3 Fig — Maps illustrating the distribution of environmental covariate data. The base layer of the map was retrieved from https://www.ibge.gov.br/geociencias/downloads-geociencias.html. (TIFF) [file pntd.0010019.s003.tiff]

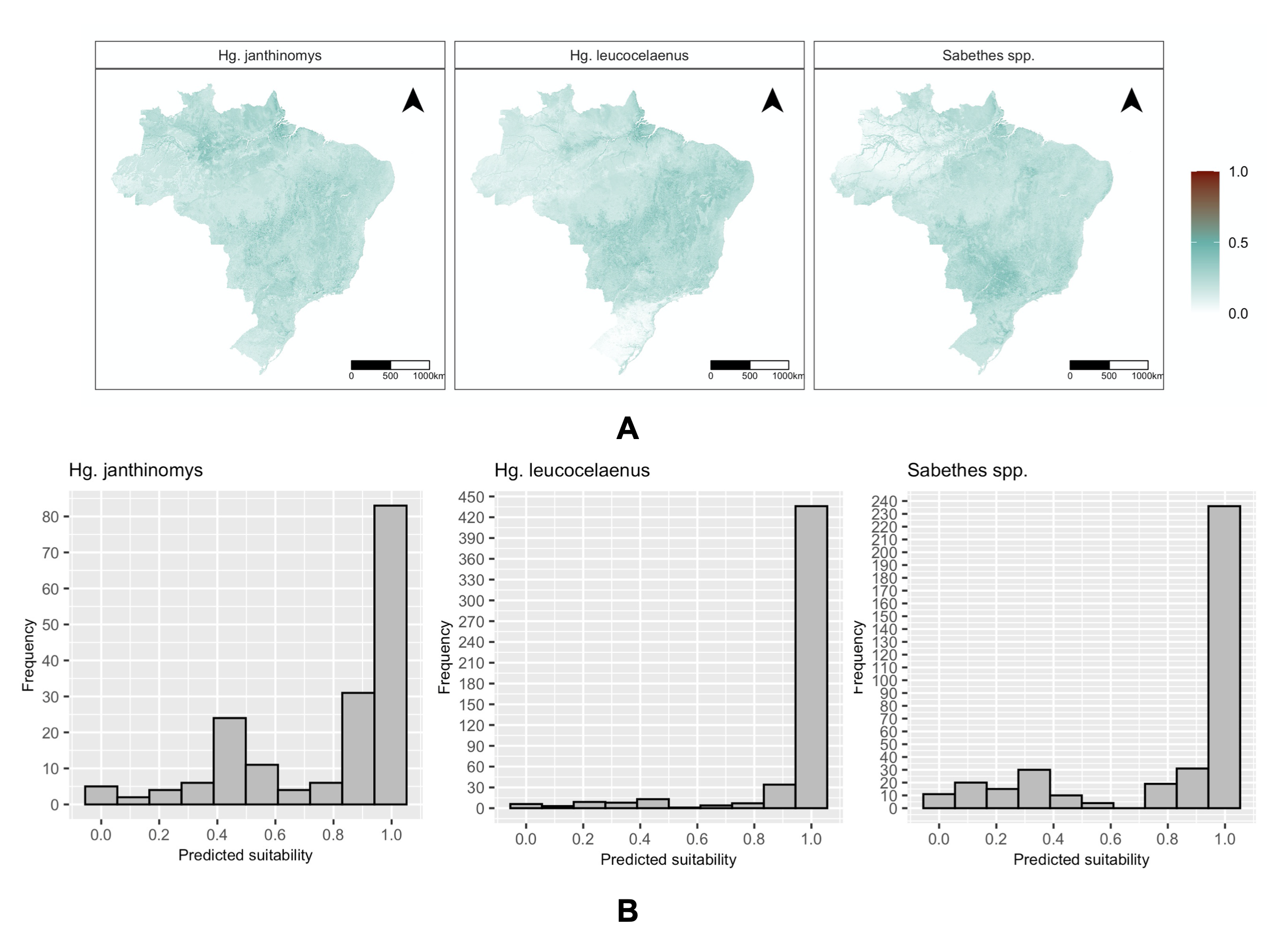

Supplement: S4 Fig — (A) Maps of prediction uncertainty calculated based on the 95% confidence interval and (B) frequency of presence occurrence points falling in each predicted range of suitability. The base layer of the map was retrieved from https://www.ibge.gov.br/geociencias/downloads-geociencias.html. (TIFF) [file pntd.0010019.s004.tiff]

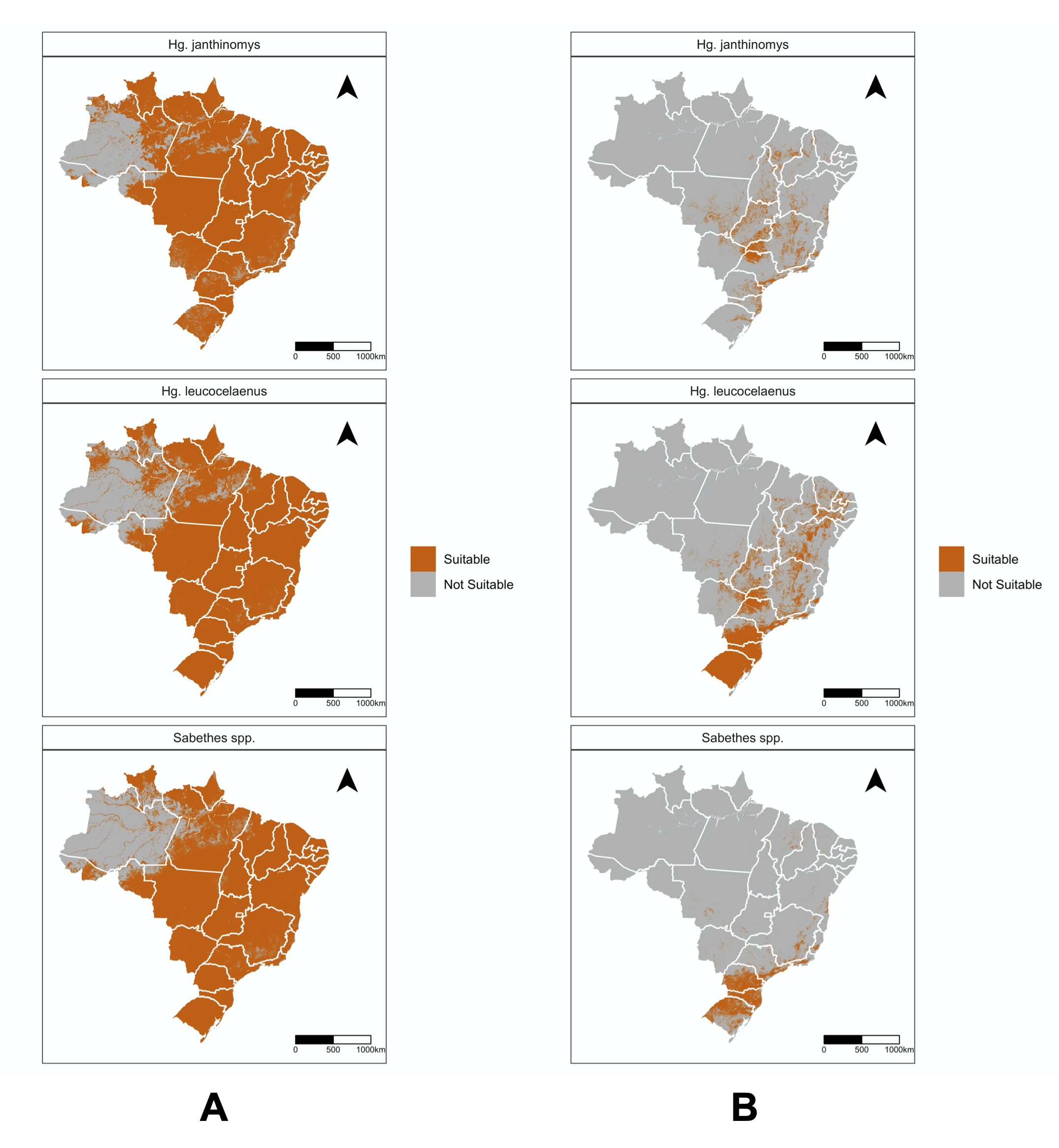

Supplement: S5 Fig — Maps showing suitable regions for Haemagogus and Sabethes spp. when environmental suitability threshold is set to (A) ≥ 0.25 and (B) ≥ 0.75. The base layer of the map was retrieved from https://www.ibge.gov.br/geociencias/downloads-geociencias.html. (TIFF) [file pntd.0010019.s005.tiff]
