# Supplementary material for: Mapping environmental suitability of Haemagogus and Sabethes spp. mosquitoes to understand sylvatic transmission risk of yellow fever virus in Brazil
Source: PLoS Negl Trop Dis. 2022 Jan 7;16(1):e0010019. doi: 10.1371/journal.pntd.0010019 (PMC8797211; doi:10.1371/journal.pntd.0010019)
Supplement: S1 Table — Metadata of environmental and land-cover covariates used in stacked binomial regression model. (DOCX) [file pntd.0010019.s007.docx]

Table S1. Metadata of environmental and land-cover covariates used in stacked binomial regression model

| **Name** | **Source** | **Units** | **Spatial Resolution (Spatial Range)** | **Temporal Range** | **Use** |
| --- | --- | --- | --- | --- | --- |
| Land Surface Temperature | MODIS (1) | Kelvin*50 | 1 km | 2001- 2019 | 3 variables; Calculate yearly min, mean, and maximum temperature over time range. |
| Precipitation | ERA5 (European Centre for Medium-Range Weather Forecasts) (2) | Mm/day | 1 km | 2001-2019 | 3 variables: Yearly total precipitation, precipitation of the driest month in the year, precipitation of the wettest month in the year averaged between 2001-2019. |
| Relative Humidity | Messina et al. 2019 (3) | %*10 | 1 km | 2015 | Monthly aggregated to yearly. |
| Elevation | [Amatulli et al. 2018](https://www.earthenv.org/topography) (4) | m | 1 km | Static | Minimum elevation in metres above sea level. |
| Gross Forest Cover Loss | [Hansen et al. 2013](https://earthenginepartners.appspot.com/science-2013-global-forest/download_v1.6.html) (5) | 0 (no loss) or 1-17 (loss in year 2001-2019) | 30 m | 2000-2019 | Forest loss during the period 2000–2019, defined as a stand-replacement disturbance, or a change from a forest to non-forest state. |
| EVI | MODIS (1) | -1 to 1 | 1 km | 16 days (2000-2016) | Mean annual EVI averaged over time period |
| Population Density | WorldPop (6) | # of people/pixel | 1 km | 2019 | Number of people per pixel, adjusted to match country total from the official UN 2019 population estimates, by the pixel surface area |
| Consensus Landcover | [Tuanmu et al. 2018](https://www.earthenv.org/landcover)  (7) | Consensus prevalence in % | 1 km | Static | 1. Evergreen Broadleaf Trees 2. Deciduous Broadleaf Trees 3. Cultivated and Managed Vegetation |
