# Supplementary material for: Mapping environmental suitability of Haemagogus and Sabethes spp. mosquitoes to understand sylvatic transmission risk of yellow fever virus in Brazil
Source: PLoS Negl Trop Dis. 2022 Jan 7;16(1):e0010019. doi: 10.1371/journal.pntd.0010019 (PMC8797211; doi:10.1371/journal.pntd.0010019)
Supplement: S1 Appendix — List of occurrence data downloads (with DOI) from Global Diversity Information Facility (GBIF). (DOCX) [file pntd.0010019.s008.docx]

**S1 Appendix**

**List of occurrence data downloads (with DOI) from Global Diversity Information Facility (GBIF)**

***Haemagogus spp.***

GBIF.org (12 January 2022) GBIF Occurrence Download <https://doi.org/10.15468/dl.n8jp98>

GBIF.org (12 January 2022) GBIF Occurrence Download <https://doi.org/10.15468/dl.jzfbmc>

***Sabethes spp.***

GBIF.org (12 January 2022) GBIF Occurrence Download <https://doi.org/10.15468/dl.svrjcc>

GBIF.org (12 January 2022) GBIF Occurrence Download <https://doi.org/10.15468/dl.dkn6d8>

GBIF.org (12 January 2022) GBIF Occurrence Download <https://doi.org/10.15468/dl.r8ndn6>

GBIF.org (12 January 2022) GBIF Occurrence Download <https://doi.org/10.15468/dl.ru723m>

GBIF.org (12 January 2022) GBIF Occurrence Download <https://doi.org/10.15468/dl.y5xtdt>

GBIF.org (12 January 2022) GBIF Occurrence Download <https://doi.org/10.15468/dl.h3dc5e>

GBIF.org (12 January 2022) GBIF Occurrence Download <https://doi.org/10.15468/dl.p48bpt>

GBIF.org (12 January 2022) GBIF Occurrence Download <https://doi.org/10.15468/dl.4xz756>

GBIF.org (12 January 2022) GBIF Occurrence Download <https://doi.org/10.15468/dl.enevaq>

GBIF.org (12 January 2022) GBIF Occurrence Download <https://doi.org/10.15468/dl.f9x2tb>

GBIF.org (12 January 2022) GBIF Occurrence Download <https://doi.org/10.15468/dl.fqshk7>

GBIF.org (12 January 2022) GBIF Occurrence Download <https://doi.org/10.15468/dl.smp9zb>

GBIF.org (12 January 2022) GBIF Occurrence Download <https://doi.org/10.15468/dl.n3589q>

GBIF.org (13 January 2022) GBIF Occurrence Download <https://doi.org/10.15468/dl.t7dmqz>

GBIF.org (13 January 2022) GBIF Occurrence Download <https://doi.org/10.15468/dl.dwxt9w>

***Culex spp.***

GBIF.org (13 January 2022) GBIF Occurrence Download <https://doi.org/10.15468/dl.r8en56>

***Limatus spp.***

GBIF.org (13 January 2022) GBIF Occurrence Download <https://doi.org/10.15468/dl.dva6as>

***Wyeomyia spp.***

GBIF.org (13 January 2022) GBIF Occurrence Download <https://doi.org/10.15468/dl.q3t5uh>

***Psorophora spp.***

GBIF.org (13 January 2022) GBIF Occurrence Download <https://doi.org/10.15468/dl.5m6sjt>

***Orthopodomyia spp.***

GBIF.org (13 January 2022) GBIF Occurrence Download <https://doi.org/10.15468/dl.gg36fp>

***Mansonia spp.***

GBIF.org (13 January 2022) GBIF Occurrence Download <https://doi.org/10.15468/dl.r5tn3q>

***Coquillettidia spp.***

GBIF.org (13 January 2022) GBIF Occurrence Download <https://doi.org/10.15468/dl.sw8yq9>
